# Classic swine fever virus NS2 protein leads to the induction of cell cycle arrest at S-phase and endoplasmic reticulum stress

**DOI:** 10.1186/1743-422X-7-4

**Published:** 2010-01-11

**Authors:** Qing-hai Tang, Yan-ming Zhang, Li Fan, Gang Tong, Lei He, Chen Dai

**Affiliations:** 1 College of Veterinary Medicine, Northwest A & F University, Yangling, Shaanxi 712100, China

## Abstract

**Background:**

Classical swine fever (CSF) caused by virulent strains of Classical swine fever virus (CSFV) is a haemorrhagic disease of pigs, characterized by disseminated intravascular coagulation, thrombocytopoenia and immunosuppression, and the swine endothelial vascular cell is one of the CSFV target cells. In this report, we investigated the previously unknown subcellular localization and function of CSFV NS2 protein by examining its effects on cell growth and cell cycle progression.

**Results:**

Stable swine umbilical vein endothelial cell line (SUVEC) expressing CSFV NS2 were established and showed that the protein localized to the endoplasmic reticulum (ER). Cellular analysis revealed that replication of NS2-expressing cell lines was inhibited by 20-30% due to cell cycle arrest at S-phase. The NS2 protein also induced ER stress and activated the nuclear transcription factor kappa B (NF-κB). A significant increase in cyclin A transcriptional levels was observed in NS2-expressing cells but was accompanied by a concomitant increase in the proteasomal degradation of cyclin A protein. Therefore, the induction of cell cycle arrest at S-phase by CSFV NS2 protein is associated with increased turnover of cyclin A protein rather than the down-regulation of cyclin A transcription.

**Conclusions:**

All the data suggest that CSFV NS2 protein modulate the cellular growth and cell cycle progression through inducing the S-phase arrest and provide a cellular environment that is advantageous for viral replication. These findings provide novel information on the function of the poorly characterized CSFV NS2 protein.

## Background

Classical swine fever (CSF) is a highly contagious and often fatal disease of pigs and is classified by the World Organization for Animal Health (OIE) as a notifiable (previously List A) disease due to its potential for rapid spread across national borders and the considerable socio-economic impact on the pig industry [1]. The causative agent of CSF is Classical swine fever virus (CSFV), which is classified as a member of the *Pestivirus *genus within the *Flaviviridae *family of viruses, accompanied by the genera Flavivirus and Hepacivirus (Hepatitis C viruses; HCV) (Lackner, Muller et al. 2004). CSFV contains a 12.3 kb positive-sense, single-stranded RNA genome that consists of 5' and 3' non-translated regions (NTR) flanking a large open reading frame that encodes a polyprotein of approximately 3898 amino acids. The polyprotein is processed into 12 mature proteins, namely, N^pro^, C, E^rns^, E1, E2, p7, NS2, NS3, NS4A, NS4B, NS5A and NS5B and at least two precursor proteins, E2-p7 and NS2-3 have been characterized [2-4].

In recent years, the functions of CSFV proteins such as N^pro^, NS3 and NS5B have been studied extensively. However, the nonstructural NS2 protein has been thought to function only as an NS2/NS3 auto-protease essential for high productivity of CSFV *in vivo *[3,5,6]. Moulin and coworkers have previously demonstrated that CSFV requires uncleaved NS2-3 and NS4A for infectious particle formation but concluded that NS2 protein alone had no essential function [6].

The N-terminus of NS2 is generated by cellular signal peptidases and the protein remains associated with intracellular membranes, presumably at the ER. The N-terminal half of NS2 is highly hydrophobic and is likely to be involved in membrane association. However, despite this information, the subcellular localization, membrane topology and protein structure have not been determined and may be due to its biochemical properties and its toxicity when expressed in bacteria [5].

Studies of other Flavivirus NS2 proteins have determined alternative functions that aid viral replication. Recently, it was reported that the HCV NS2 protein inhibits cellular proliferation by the induction of cell cycle arrest at S-phase through the down-regulation of cyclin A expression [7]. The S-phase of the cell cycle can provide a cellular environment that is beneficial for viral replication. There is evidence from other viruses, such as herpes viruses, that the evolution of viral proteins that regulate the host cell cycle provides a replicative advantage [8-10]. The human T-lymphotrophic virus type 1, a retrovirus, encodes a Tax oncoprotein that promotes entry of host cells into S-phase and blocks mitosis [11].

It is from these parallels that we hypothesize that the CSFV NS2 protein has properties that can alter cell cycle replication. Presently, no data exists on the subcellular localization of CSFV NS2 protein and its effects on cell growth and cell cycle progression. The swine endothelial vascular cell is one of the CSFV target cells, vascular endothelial cells maintain the haemostatic balance by providing a quiescent, anti-thrombotic barrier [12]. This present study was initiated to demonstrate the subcellular localization of CSFV NS2 protein and elucidate the effects and mechanisms of this protein on cell growth and cell cycle progression. Here, we show that the expression of NS2 protein causes cell growth retardation in swine umbilical vein endothelial cell line (SUVEC) established in our lab previously [13] and increased the proportion of the cells in the S-phase with a concomitant decrease in the proportion of cells in the G0/G1 phase. The cell cycle effects were associated with the activation of NF-κB and the rapid degradation of cyclin A but not the down-regulation of cyclin A transcription. Furthermore, we show that CSFV NS2 protein localized in ER and induced ER stress. The results of these findings have potentially important implications for understanding the molecular mechanisms of pathogenesis for this economically important agricultural disease.

## Materials and methods

### Vectors, virus and cell culture

The pEGFP-C1 and pEGFP-N1 eukaryotic expression vector were purchased from Clontech (USA) and competent *Escherichia coli *DH5α used for cloning were purchased from Tiangen Biotech (China). Virulent CSFV (Shimen strain) was obtained from the Control Institute of Veterinary Bioproducts and Pharmaceuticals (China) and propagated in PK-15 cells. The established swine umbilical vein endothelial cell line, SUVEC, was cultured as previously described [13]. Briefly, SUVEC were cultured at 37°C and 5% CO_2 _in M199 (Gibco, UK) medium containing 20% foetal calf serum (Hyclone, China), 50 μg/mL heparin (Sigma-Aldrich, USA), 20 μg/mL unrefined hypothalamus-pituitary supernatant (produced from sheep in this laboratory), and 100 μg/mL penicillin/streptomycin. The culture medium was replaced every 3 days. Porcine kidney cells (PK-15; ATCC CCL-33) were grown in Dulbecco's modified eagle medium (DMEM; Gibco, UK), supplemented with 10% foetal calf serum (Hyclone, China).

### Antibodies and reagents

Mouse anti-GFP monoclonal antibody (mAb), horseradish peroxidase-conjugated goat anti-rabbit and horseradish peroxidase-conjugated goat anti-mouse antibodies were purchased from Millipore (USA). The anti-porcine cyclin A rabbit polyclonal antiserum was prepared by our laboratory. The mouse anti-porcine GAPDH antibody was obtained from LifeSpan Biosciences (USA). The MG132 proteasome inhibitor was purchased from Calbiochem (USA) and the nuclear staining dye Hoechst 33342 and ER-Tracker™ Red probe were obtained from Invitrogen (USA).

### Plasmid construction and transfection

Primers NS2R (5'-CCCATAGTGTCACATACCAG-3'), F1 (5'-GAAGTCGACGGAAAGATAGATGGCGGTTGGCAGC-3') (the underlined sequences are the *Sal *I restriction enzyme recognition sites), R1 (5'-GAAGGATCCTCTAAGCACCCAGCCAAGGTGTTCCA-3') (the underlined sequences are the *Bam*H I restriction enzyme recognition sites), F2 (5'-GAAAAGCTTGGAAAGATAGATGGCGGTTGGCAGC-3') (the underlined sequences are the *Hind *III restriction enzyme recognition sites), R2 (5'-GAACCGCGGTCTAAGCACCCAGCCAAGGTGTTCCA-3') (the underlined sequences are the *Sac *II restriction enzyme recognition sites), were designed to amplify the CSFV NS2 gene according to the archived CSFV Shimen strain nucleotide sequence (GenBank: AF092448). Primer NS2R was used for the first cDNA strand synthesis and the primers F1 and R1 were used for the PCR amplification and were designed with 5' terminal restriction enzyme recognition sites for aid cloning into pEGFP-C1, F2 and R2 were also used for the PCR amplification and were designed with 5' terminal restriction enzyme recognition sites for aid cloning into pEGFP-N1. Primers were synthesized by Shanghai Invitrogen (China). In order to synthesize CSFV NS2 cDNA, total RNA was extracted from PK-15 cells infected with CSFV Shimen strain using Trizol Reagent (Invitrogen, USA) and reverse transcribed using a first-strand cDNA synthesis kit (Takara Bio., Dalian China). The PCR was performed in a total volume of 25 μL using a thermocycling protocol of 94°C for 4 min, 94°C for 30 s, 60°C for 30 s and 72°C for 1.5 min for 35 cycles, followed by 72°C for 10 min. The RT-PCR product was detected by 1.0% agarose gel electrophoresis, purified from the gel and digested with restriction enzymes to be cloned into the pEGFP-C1 and pEGFP-N1 expression vector. The resulting recombinant plasmid, named pEGFP-NS2 and pNS2-EGFP, was recovered from transformed *E. coli *using a plasmid mini-kit (Axygen, China) and identified by restriction enzyme digestion and sequence analysis.

SUVEC cells were seeded into 12-well dishes 24 h before being transfected (up to 60-70% confluence). Cells were transfected with pEGFP-NS2, pNS2-EGFP and pEGFP-C1 control vector by Lipofectamine 2000 (Invitrogen, USA) and passaged (up to 80% confluence) in selection media containing 1500 μg/mL G418 for two weeks. When all control cells had evidence of death in the presence of the selection agent, cultures transfected with pEGFP-NS2, pNS2-EGFP and pEGFP-C1 were propagated for two further weeks in medium containing 400 μg/mL G418. The resulting stably transfected cell lines expressing either GFP, GFP-NS2 or NS2-GFP fusion proteins were used for subsequent analyses.

### Confocal microscopy

To examine the expression and subcellular localization of CSFV NS2 protein, the stable cell lines expressing GFP-NS2, NS2-GFP protein or control cells (GFP and untransfected cells) were grown on glass coverslips in 6-well tissue culture plates. After pretreatment with 2.5 μM MG132 proteasome inhibitor (Calbiochem, USA) for 16 h, cells were washed with Hank's balanced salt solution (HBSS) and incubated with Hoechst33342 at 37°C for 15 min, and then washed twice with HBSS, after that, all the cells were incubated with ER-Tracker™ Red probe (Invitrogen, USA) at 37°C for 30 min. Cells were subsequently washed with DMEM without serum and images were viewed by laser confocal scanning microscopy (Model LSM510 META, Zeiss, Germany) and the images were processed by Adobe Photoshop software.

### Western blot

Whole cell extracts were prepared by washing cells with PBS, harvested by scraping and then suspended in 1 mL PBS. Following centrifugation, the cells were resuspended in cell lysis buffer (50 mM Tris-HCl, 5 mM EDTA, 150 mM NaCl, 0.1% NP-40, 0.5% deoxycholic acid, 1 mM sodium orthovanadate, 100 μg/mL PMSF and protease inhibitors) and centrifuged at 15 000 × g for 30 min at 4°C. Cell extracts were resolved by 12% sodium dodecyl sulphate polyacrylamide gel electrophoresis (SDS-PAGE) and transferred to a PVDF membrane (Millipore, USA). The membrane was blocked overnight with 5% skim milk in TNT buffer (20 mM Tris-HCl [pH 7.5], 150 mM NaCl, and 0.05% Tween 20) and then incubated with mouse anti-GFP-tag mAb, or the anti-swine cyclin A rabbit polyclonal antiserum for 2 h. Porcine GAPDH proteins were detected using mouse anti-porcine GAPDH antibody. Detection of primary antibodies was performed with either horseradish peroxidase-conjugated goat anti-rabbit antibody or horseradish peroxidase-conjugated goat anti-mouse antibody, as appropriate. The protein bands were visualized by enhanced chemiluminescence methods as per the manufacturer's instructions (Millipore, USA).

### Cell proliferation assay

The MTS cell proliferation assay was performed to determine the growth properties of CSFV NS2-expressing and control cells according to the manufacturer's instructions. Briefly, cells were seeded in 96-well culture plates at a concentration of 4 × 10^3 ^cells/well in 100 μL culture medium. After incubation at 37°C for 24, 48, 72 and 96 h, the culture medium was carefully replaced with 100 μL of a fresh medium without disturbing the cells. Twenty microlitres of MTS (Promega, USA) reagent was added to each well and incubated in a CO_2 _incubator at 37°C for 4 h. The absorbance at a wavelength of 492 nm was read on a microplate reader (Model 680, Bio-Rad, USA) at appropriate time intervals.

### Cell cycle analysis by flow cytometry

Since DNA fragmentation suggests apoptotic DNA damage and indicated by a distinct sub-G0/G1 peak in flow cytometry assay [14], the cell cycle and apoptosis was measured as previously described [15]. In brief, approximately 2 × 10^6 ^cells of the stable cell lines and control cells were trypsinised and collected. Following two washes with PBS, cells were resuspended in 70% ethanol and fixed at 4°C for 18 h. Cells were washed and resuspended in PBS containing 20 μg/mL of RNase A and 50 μg/mL of propidium iodide (PI) and incubated on ice for 30 min. Finally, the nuclear DNA content was determined using a Coulter Epics XL flow cytometer (Beckman Coulter, USA).

### Quantitative real-time RT-PCR for porcine cyclin A and GRP78

Since the sequence of porcine cyclin A mRNA has not been previously published, it was necessary to determine this sequence for the development of the quantitative real-time RT-PCR assay for porcine cyclin A mRNA. Two primers were designed to amplify the human cyclin A nucleotide sequences (GenBank: X51688) and used to amplify the porcine cyclin A gene by RT-PCR given that significant homology is likely to exist between the two species in this gene. The sequences of these primers were as follows: R: 5'-GATTTACATCTTAGAAAACAAAGG-3'; PC1: 5'-GGTGATCCCGCCGTCCACT-3' and PC2: 5'-GATTTACATCTTAGAAAACAAAGGCAGTC-3'. Total RNA was extracted from PK-15 cells and reverse transcribed using a first-strand cDNA synthesis kit (Takara, Japan). The PCR reaction was performed in a total volume of 25 μL using a thermocycling protocol of 94°C for 4 min, 94°C for 30 s, 62°C for 30 s and 72°C for 1.5 min for 35 cycles, followed by 72°C for 10 min. The PCR product was purified and cloned into the pMD-19T vector (Takara, Dalian China) and positive recombinant plasmids were identified by restriction enzyme digestion and sequenced. The sequences of porcine cyclin A were submitted to GenBank, and are available under the accession number, GQ265874. To quantify cyclin A mRNA, total RNA was extracted from cells using Trizol reagent (Invitrogen, USA) according to the manufacturer's instructions. Synthesis of cDNA was performed with 2 μg of total RNA in a 25 μL reaction containing 20 U/μL M-MuLV reverse transcriptase (Takara Bio., China), 0.5 μM dNTP and 0.25 μM Oligo(dT)18 primer at 42°C for 3 h. Real-time PCR of cDNA was carried out using the SYBR real-time PCR kit (Takara Bio., Dalian China) on a real-time PCR system (model 7500; ABI, USA) with the following cycling profile: 5 min at 95°C followed by 40 cycles of 10 s at 95°C and 20 s at 60°C. The individual samples were normalized for genome equivalents using the respective CT value for the porcine β-actin housekeeping gene. One pair of primers was designed for the quantification of porcine cyclin A mRNA levels by real-time PCR according to the obtained porcine cyclin A sequences. The sequences for the two primers were PC1: 5'-AAGTTTGATAGATGCTGACCCGTAC-3' and PC2: 5'-GCTGTGGTGCTCTGAGGTAGGT-3'. Additionally, primers for detecting the porcine β-actin housekeeping gene (GenBank: DQ845171 and SSU07786), ActinF (5'-CAAGGACCTCTACGCCAACAC-3') and ActinR (5'-TGGAGGCGCGATGATCTT-3') were synthesized.

To analyze the expression of glucose regulated protein, 78 kDa (GRP78), a well characterized ER chaperone protein that is a marker of ER stress, we developed a real-time PCR assay for its detection [16-21]. A pair of primers, GRP78F (5'-AATGGCCGTGTGGAGATCA-3') and GRP78R (5'-GAGCTGGTTCTTGGCTGCAT-3'), were designed according to the available porcine GRP78 sequences (GenBank: X92446) to detect the porcine GRP78 mRNA level in cells.

### Quantification of NF-κB p50 DNA-binding activity

To determine the alteration of NF-κB activity by GFP, GFP-NS2 and NS2-GFP proteins in the established cell lines, the level of p50 activity was measured using the NF-κB TransAM kit (Active Motif) according to the manufacturer's instructions. Briefly, cells nuclear extraction was prepared by using the Nuclear Extract Kit (Active Motif) and protein concentrations were measured using the Bradford assay (Bio-Rad). Lysates (20 mg total proteins) were incubated in ELISA wells coated with the oligo-nucleotide motif recognized by active p50, p50 was then detected using a specific antibody, followed by a secondary antibody conjugated to peroxidase. The colorimetric reaction was measured at A450, with 620 nm acting as the reference wavelength. This experiment was repeated three times.

### Statistical analysis

SPSS^®^15.0J (SPSS Inc, USA) was used to perform the statistical analyses including one-way analysis of variance (ANOVA) followed by Dunnett's test.

## Results

### Expression and subcellular localization of CSFV NS2 protein

**Figure 1:**
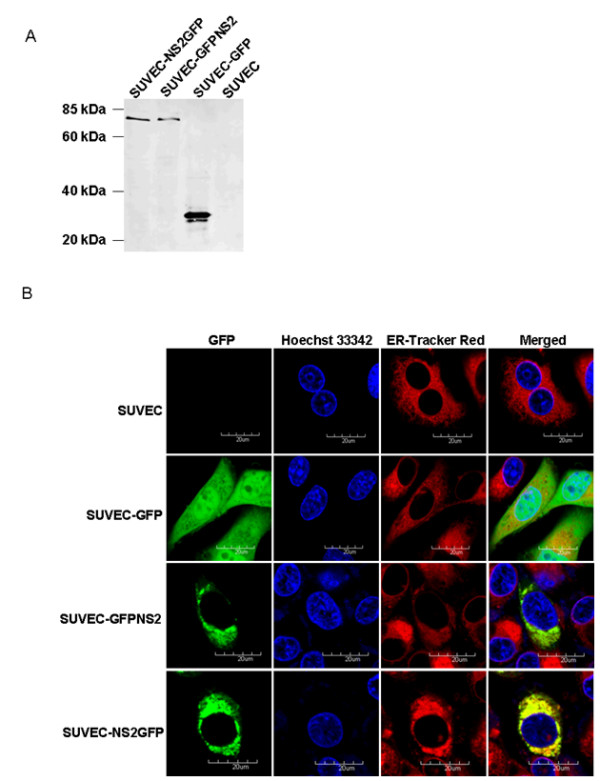
**Detection and subcellular localization of the GFP-NS2 and NS2-GFP fusion protein expressed in SUVEC**. (A) Western immunoblot using anti-GFP antibody of cellular proteins isolated from various cell lines. (B) Confocal microscopy images of SUVEC cells. All the cell lines were stained by Hoechst33342 and ER-Tracker™ Red. Merged images show co-localization of GFP, GFP-NS2 and NS2-GFP in the ER. Bar = 20 μm for all the figures.

The expression and subcellular localization of CSFV NS2 protein in SUVEC stable cell lines were analyzed. Western blot analysis showed that the GFP-NS2 and NS2-GFP fusion protein had a molecular weight of approximately 80 kDa and was present in all cells transfected with the pEGFP-NS2 plasmid and pNS2-EGFP plasmid, and no signal can be detected from the negative SUVEC control cells (Fig. 1A). Since the molecular weight of GFP is known to be approximately 27 kDa, the molecular weight of the putative CSFV NS2 protein is approximately 53 kDa. The subcellular localization of NS2 was investigated by confocal fluorescence microscopy and showed that GFPNS2 protein and NS2GFP protein were both distributed in the endoplasmic reticulum, by contrast, the GFP protein was distributed in the whole cells (Figs. 1B). These results revealed that CSFV NS2 protein localized to the ER, the report prtein GFP which either fused to the -NH2 terminal or the -COOH terminal of CSFV NS2 protein did not affect the trans-localization of CSFV NS2 protein.

### Inhibition of cell proliferation by CSFV NS2 protein

**Figure 2:**
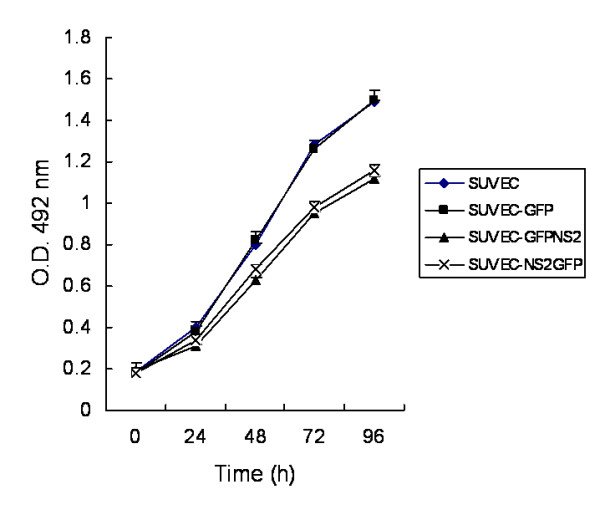
**Cell proliferation assays of stable NS2-expressing cell lines**. The MTS assay was used to measure proliferation of 4 × 10^3 ^cells from SUVEC cell lines over time. Each data set represents the mean ± S.D. of six replicates.

Compared with control cells (pEGFP-C1 transfected and untransfected), the CSFV NS2 expressing cells divided much more slowly leading to a significantly decreased cell number after a period of time. Cell proliferation of NS2 expressing cells measured by the MTS assay was decreased by approximately 20 - 30% over a time course of 72 to 96 h (Fig. 2).

### CSFV NS2 protein induces cell cycle arrest at S-phase

**Figure 3:**
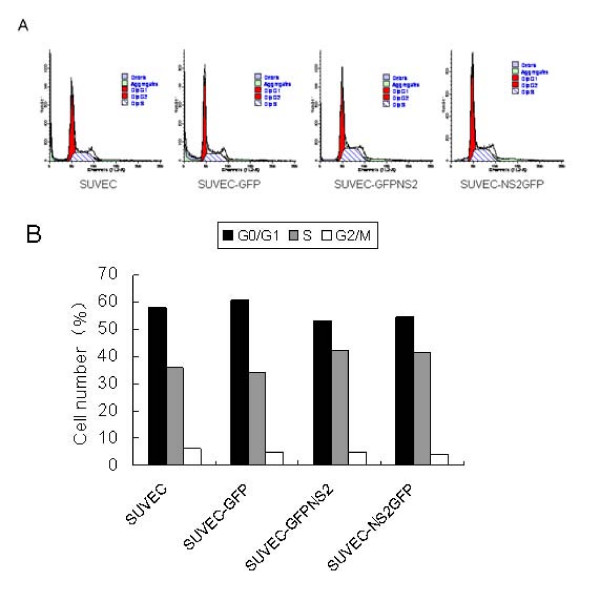
**The analysis of the stages of cell division of expressing CSFV NS2 protein by flow cytometry**. (A) Histograms from flow cytometry data for propidium iodide staining. (B) Analysis of the percentage of cells in each phase of the cell cycle from flow cytometry data.

To determine whether the growth inhibition of CSFV NS2-expressing cells was due to the arrest of the cell cycle at a certain phase(s) of cell division, flow cytometric analysis was performed based on DNA content in nuclei stained with PI. The proportions of G0/G1 phase, S-phase and G2/M phases for the control cells were 57.96%, 35.98% and 6.06%, respectively. For SUVEC expressing GFP, the proportions of the phases were G0/G1: 60.84%, S-phase: 34.19%, and G2/M: 4.96%, whereas for GFP-NS2-expressing SUVEC stable cells, the proportions were G0/G1: 52.96%, S-phase: 42.29% and G2/M: 4.76%, consistently, for the NS2-GFP-expressing SUVEC stable cells, the proportions were G0/G1: 54.40%, S-phase: 41.40% and G2/M: 4.19% (Fig. 3A). Apoptosis was also analyzed by flow cytometry but no differences were observed between untransfected cells or pEGFP-C1 transfected cells and cells expressing GFP-NS2 or NS2-GFP fusion protein. A sub-G0/G1 peak was not detected by flow cytometry for both the GFP-NS2-expressing, NS2-GFP-expressing and control cells and suggests that the CSFV NS2 protein induced cell cycle arrest in the S-phase, rather than inducing apoptosis. The results showed that relative to control cells, NS2 expression causes a significant increase in the proportion of cells in the S-phase accompanied by a decrease in the cell proportion in the G0/G1 phase (Fig. 3B). Taken together, these results strongly suggest that NS2 protein causes the inhibition of cell growth by the induction of cell cycle arrest in the S-phase.

### CSFV NS2-induced cell cycle arrest is associated with proteasomal degradation of cyclin A

**Figure 4:**
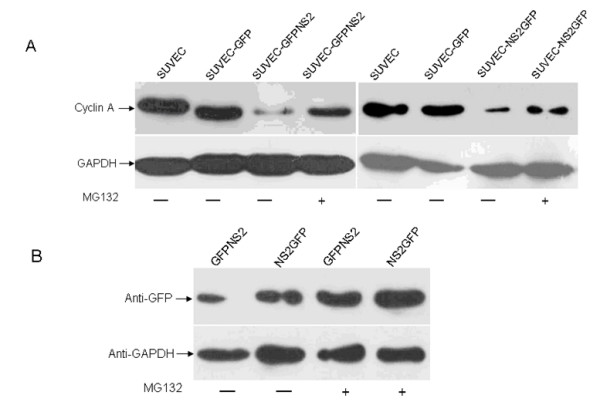
**The effect of CSFV NS2 expression on the porcine cyclin A protein level**. (A) The level of cyclin A expression was determined by western blot with the anti-porcine cyclin A rabbit polyclonal antiserum in all cell lines treated or untreated with MG132. (B) The expression of NS2 from the same samples with and without the treatment of MG132 was detected by using anti-GFP mAb.

**Figure 5:**
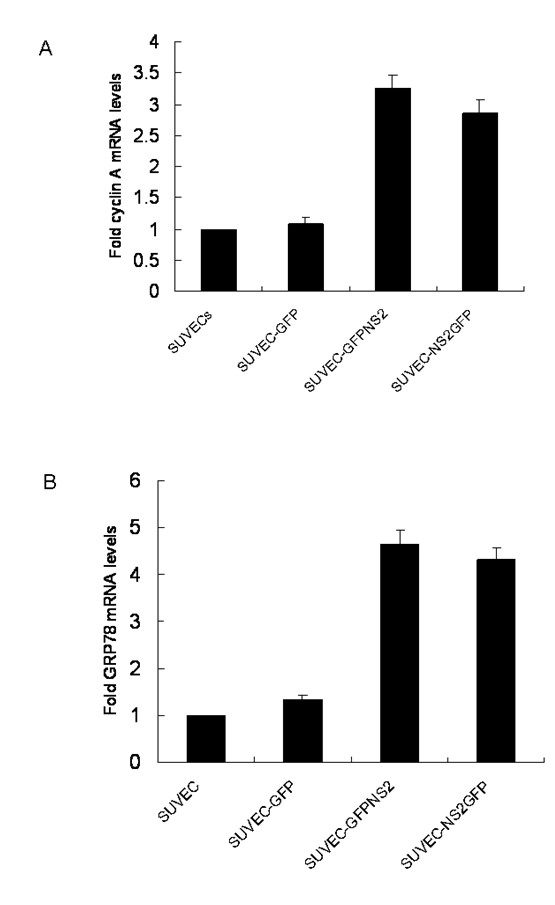
**The effect of CSFV NS2 expression on porcine cyclin A and GRP78 transcription in cultured SUVEC cells**. Total RNA was extracted from cells expressing either GFP alone, GFP-NS2 fusion, NS2-GFP fusion or untransfected cells. Real-time RT-PCR analysis of (A) cyclin A and (B) GRP78 mRNA levels were normalized to the corresponding C_T _value for porcine β-actin mRNA. The basal expression level in untransfected controls was assigned a value of 1 for each experiment.

**Figure 6:**
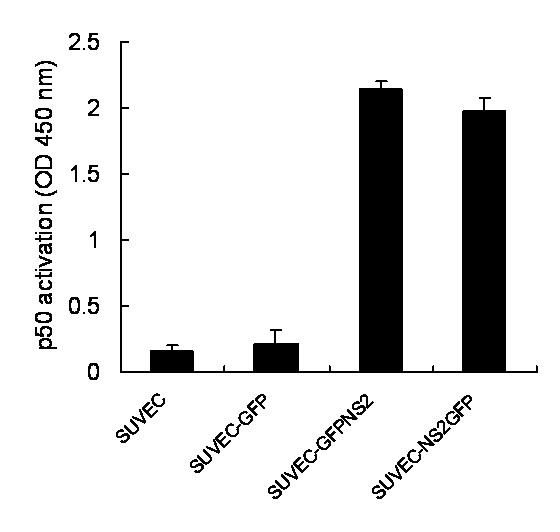
**The effect of CSFV NS2 expression on NF-κB p50 DNA-binding activity in SUVEC cell lines**. NF-κB p50 activation was determined using the TransAM assay. The experiment was repeated three times and the figure shows a representative experiment.

Since cyclin A is required for the entry into G2/M phase, the cyclin A protein levels in NS2 expressing cell lines and control cells were analyzed by western blot assay. Cyclin A expression level was significantly decreased in both cell lines that expressed NS2 protein compared with control cells. Furthermore, when the GFP-NS2-expressing and NS2-GFP-expressing cell lines were pre-treated with MG132, blocking proteasomal degradation of proteins, the relative intensity of cyclin A western blot signal was significantly increased (Fig. 4), suggesting that cyclin A is rapidly degraded in the presence of NS2. To further support these findings, quantitative real-time RT-PCR was employed and showed that cyclin A mRNA levels in the GFP-NS2-expressing and NS2-GFP-expressing cell lines were significant higher than in control cells (Fig. 5A). This suggests that the induction of cell cycle arrest in the S-phase by CSFV NS2 protein is associated with the increase of cyclin A proteasomal degradation rather than a decrease of cyclin A transcription. Moreover, transcription of GRP78, the ER molecular chaperone was also increased in GFP-NS2-expressing cell lines compared with controls (Fig. 5B). Analysis of the activity of NF-κB in GFP-NS2-expressing and NS2-GFP-expressing cell lines using a TransAMTM NF-κB p50 Transcription Factor Assay Kit demonstrated that the NF-κB was significantly activated compared with control cells (Fig. 6). Together, these analyses have shown that expression of CSFV NS2 results in the up-regulation of GRP78, cyclin A and NF-κB suggesting a role of NS2 in ER stress activation. Additionally, NS2 induces cell cycle arrest at the S-phase and is associated with the increased proteasomal degradation of cyclin A.

## Discussion

Recent years, many studies were focus on the the function of an NS2/NS3 auto-protease, the function of uncleaved NS2-3 on the pestivirus virion assembly [2-6,22,23]. Despite this vast amount of research, the subcellular localization and function of the CSFV NS2 protein is still unclear. Previous studies have presumed that the CSFV p7 protein assists NS2 localization by forming a leader sequence that properly orients NS2 in the ER membrane [3,5]. However, until now, no experimental data on CSFV NS2 protein membrane topology or protein structure has been available. Furthermore, the function of this protein is yet to be determined, particularly with regard to its effect on host cell physiological changes. In this study, we constructed an expression vector and generated stably expressing cell lines of CSFV NS2 in fusion with the GFP protein that allowed the analysis of many of these properties. Co-localization studies clearly showed that GFP-NS2 and NS2-GFP localized in the ER. Moreover, the results of the bioinformatics analysis showed that N-terminal half of CSFV NS2 is highly hydrophobic involved in membrane association (data not show). Recently, Yamaga and colleagues (2002) demonstrated that the membrane-association of HCV NS2 is p7-independent and this protein contains at least two internal signal sequences for membrane association and likely has multiple transmembrane domains [24], confirming our findings.

In this study, the effect of CSFV NS2 protein on CSFV-target SUVEC cells proliferation was determined. Various analyses showed that the CSFV NS2 protein was able to inhibit the cell proliferation and induce cell cycle arrest at S-phase. Recently, it was reported that the HCV NS2 protein inhibited cell proliferation and induced cell cycle arrest in the S-phase in mammalian cells through inhibition of NF-κB activation and down-regulation of cyclin A expression [7]. However, in this study, the western blot analysis suggested that cyclin A protein levels were not simultaneously elevated, by contrast, the cyclin A protein levels in the CSFV NS2 protein-expressing cell lines were significantly lower than that in the control cell lines. To investigate whether the turnover rate of the cyclin A in the NS2-expressing cell lines was accelerated, the NS2-expressing cell lines were treated with the proteasome inhibitor, MG132 for 24 h. Interestingly, the cyclin A protein levels were significantly elevated in treated compared with untreated cell lines (Fig. 4). Furthermore, we also revealed that the CSFV NS2 protein significantly promoted the transcription of cyclin A through the activation of NF-κB in SUVEC cells, which consist with the previous study that CSFV infection activated the NF-κB activity in the porcine vascular endothelial cells cultured *in vitro *[25]. These results suggested that the CSFV NS2 protein played an important role not only in the activation of NF-κB that consequently increased the transcription levels of cyclin A but also in the accelerated proteasomal degradation.

The Flaviviridae family of viruses encompasses many important human pathogens, including HCV. A characteristic of Flaviviruses is their utilization of the ER as the primary site for polyprotein processing, glycoprotein biogenesis and particle assembly [26]. The ER is an organelle that has essential roles in multiple cellular processes that are required for cell survival and normal cellular functions [27]. Viruses that use the ER as an integral part of their replication strategy must contend with the ER stress response and the downstream consequences of ER stress signalling. Recently, it was reported that HCV NS2 protein activated endoplasmic reticulum stress signalling, HCV NS2 expression increased phosphorylation of elF2α and up-regulated luciferase expression driven by molecular chaperone GRP78 and transcription factors ATF6, or GADD153 promoter [28].

Consistent with the characterization of the HCV NS2 protein as a membrane protein [24], our observations also show that the CSFV NS2 protein is an ER membrane protein that is likely to be responsible for inducing ER stress. In the current study, we show that the CSFV NS2 protein was found to localize in the ER and able to induce ER stress, as indicated by the significant up-regulation of the molecule chaperon GRP78, a typical marker of ER stress. Since it was demonstrated that under conditions of ER stress, mammalian cells accelerated the retrograde export of proteins from the ER to the cytosol for ubiquitylation and proteasome-mediated degradation [29-31], taken together, we speculate that the CSFV NS2-expressing cells accelerated the retrograde export of cyclin A proteins from the ER to the cytosol for ubiquitylation and proteasome-mediated degradation. Yang and colleagues have previously demonstrated that HCV NS2 protein inhibited cell proliferation and induced cell cycle arrest in the S-phase through inhibition of NF-κB activation and down-regulation of cyclin A protein expression, but failed to analyse its effect on mRNA levels [7]. The present study showed that CSFV NS2 protein employed a different mechanism to inhibit the host cells proliferation compared with the HCV NS2 protein.

## Conclusion

Taken together, the CSFV NS2 protein localized in the ER and inhibited the SUVEC cell growth and regulated division through cell cycle arrest in the S-phase. The induction of cell cycle arrest in the S-phase by CSFV NS2 protein is associated with an increase in cyclin A degradation rather than a decrease in cyclin A transcription level. The present study has provided knowledge that will enable us to further explore the membrane topology and functions of CSFV NS2 protein that have remained largely unexplored.

## Competing interests

The authors declare that they have no competing interests.

## Authors' contributions

QHT organized the whole process, took part in all the experiments and wrote the manuscript. YMZ designed the whole project. LF participated in the plasmid construction, cell transfection and confocal microscope; GT made great contribution to the MTS assay; LH and CD carried out the cell culture and Real time PCR. All authors read and approved the final manuscript.
